# Protocol for inferring mechanical stresses in tissues using ForSys, an open-source Python tool

**DOI:** 10.1016/j.xpro.2026.104595

**Published:** 2026-05-25

**Authors:** Augusto Borges, Jerónimo R. Miranda-Rodríguez, Alberto S. Ceccarelli, Guilherme Ventura, Jakub Sedzinski, Hernán López-Schier, Osvaldo Chara

**Affiliations:** 1 Department of Physiology, Development and Neuroscience, University of Cambridge, Cambridge CB2, UK; 2 Instituto de Neurobiología, Universidad Nacional Autónoma de México (UNAM), Boulevard Juriquilla, 3001 Juriquilla, Mexico; 3 Division of Science, New York University Abu Dhabi, Saadiyat Island, United Arab Emirates; 4 School of Biosciences, University of Nottingham, Sutton Bonington Campus, Nottingham LE12, UK; 5 The Novo Nordisk Foundation Center for Stem Cell Medicine (reNEW), University of Copenhagen, Copenhagen, Denmark; 6 Department of Biomedical Sciences, University of Copenhagen, Copenhagen, Denmark; 7 Research Institute for Molecular Pathology (IMP), Vienna, Austria; 8 Instituto de Tecnología, Universidad Argentina de La Empresa, Buenos Aires, Argentina

**Keywords:** Bioinformatics, Biophysics, Developmental biology, Systems biology, Tissue Engineering

## Abstract

Stress inference offers a rapid computational approach for estimating the mechanical state of a tissue without requiring invasive experiments. Here, we present a protocol for inferring mechanical stresses in tissues using ForSys, an open-source Python tool. We describe steps for preparing the Python environment, using the command-line interface, installing and using the graphical user interface (GUI), and importing data. We then detail procedures for correcting automatic connections between time points, determining scale parameters, and plotting and quantifying the inference results.

For complete details on the use and execution of this protocol, please refer to Borges et al.^1^

## Highlights

• Instructions for loading time-lapse images of membrane-tagged tissues into ForSys
• Steps for running mechanical inference via the ForSys Fiji plugin GUI
• Procedures for batch processing biological image data via command line or Python
• Guidance on interpreting and visualizing mechanical states of evolving tissues

## Abstract (editor highlights)

Publisher’s note: Undertaking any experimental protocol requires adherence to local institutional guidelines for laboratory safety and ethics.

## Graphical abstract

**Figure:**
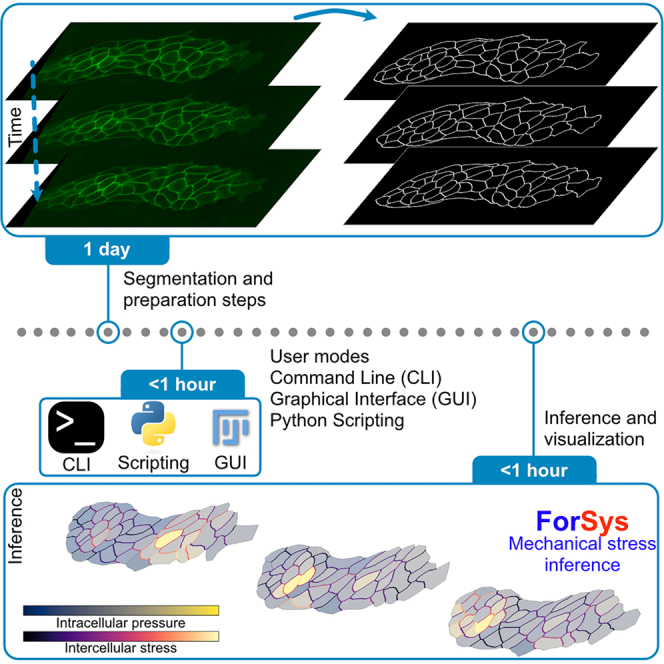

## Before you begin

Stress inference techniques are a set of methods that use the geometrical information of a tissue to infer the relationships among stresses at each cell membrane.^2^^,^^3^^,^^4^^,^^5^^,^^6^ While static stress inference uses single microcopy images, dynamic stress inference allows for inferring junctional stress in time.^1^^,^^2^^,^^7^ This protocol shows how to determine the mechanical state of a tissue from either a single microscopy image or time-lapse microscopy data using the ForSys software.^1^^,^^8^

ForSys enables the inference of intercellular stress and intracellular pressures from two-dimensional time-lapse movies of confluent tissues. The protocol presented herein will be helpful for scientists who wish to apply the stress inference techniques to microscopy images, as well as for theoreticians who would employ it on their *in silico* data.

**Figure 1:**
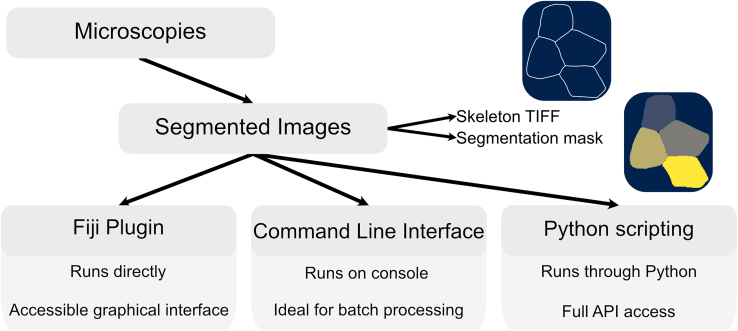
ForSys modes flowchart. ForSys accepts two input types: skeletonised TIFF images or segmentation masks. ForSys can be used through three possible modes. The Fiji plugin provides a graphical interface that enables quick, easy access to inferences. The command-line interface is ideal for batch processing because it can be integrated into any shell. Finally, ForSys can be run by importing the package into Python and running through scripting, allowing full access to its API.

ForSys can be used in three different modes, which vary in terms of ease of use and customizability. The Graphical User Interface (GUI) provides the simplest access to ForSys’s functions (Figure 1, left), while the Command Line Interface (CLI) offers more control over the parameters (Figure 1, middle). Finally, interacting with the code directly allows full access to the software’s options, but requires basic knowledge of the Python language (Figure 1, right). Figure 1 provides a flowchart of ForSys usage detailing the three usage modes and the expected inputs TIFF skeletons or segmentation masks.

We will first describe how to install the software and prepare the necessary files and folders for its use. Then, we will describe how to use the Command Line Interface to perform inferences, followed by the installation of the Graphical User Interface in Fiji, and its usage. Finally, we will demonstrate how to perform inferences using Python scripting on both synthetic and experimental data.

A prerequisite for stress inference analysis of experimental data is the availability of tissue images, which can be either single frames or time-lapse movies. Moreover, ForSys requires segmented images in the form of a skeleton (∗.tif) or segmented masks (∗.npy). The Key Resource Tables detail the software used to create the segmentation and run the inference. When following this protocol for the first time, we recommend using the experimental images located in the “examples/data/in_vivo” folder for sections “using the command line interface”, “using the graphical user interface (GUI)”, “static inference of the mechanical state” and “dynamic inference of the mechanical state”. We also recommend using the simulated data provided in the “examples/data/in_silico” folder for the *in silico* sections when following this protocol for the first time. These folders can be found in the software’s repository (https://github.com/borgesaugusto/forsys).^8^ To use the graphical interface or the command line with these files, they must be organised according to the protocol, as explained in step 7 and the note below it. Similarly, the repository’s folder “examples/data/in_silico” contains *in silico* data generated with Surface Evolver.^9^

***Note:*** All three modes require installing a Python environment where ForSys can run. The GUI option requires using the Conda environment and then installing it in Fiji.

### Innovation

The ForSys software extends the applicability of existing stress inference techniques to dynamically evolving tissue geometry.^1^^,^^7^ ForSys incorporates information on local movements into the inference pipeline, increasing the accuracy of the resulting representation of the mechanical state.

## Key resources table

**Table:** 

REAGENT or RESOURCE	SOURCE	IDENTIFIER
**Software and algorithms**		

ForSys v1.1.5	Chara lab^8^	https://doi.org/10.5281/zenodo.18387574
Epyseg v0.1.52	Prud'Homme Lab^10^	https://doi.org/10.1242/dev.194589
Tissue Analyzer	Eaton lab^11^	https://doi.org/10.1007/978-1-4939-6371-3_13

## Step-by-step method details

### Preparing the Python environment

**Timing: <30 min**

In this step, we will create and configure the environment to use the ForSys package. We recommend using Anaconda to create and manage Python environments^12^; any other method to install the ForSys package can be used. The Anaconda Navigator can be used to create and configure the environment. Please follow the Methods video S1 for a video tutorial on preparing the environment through these steps. In this example, we will install and use *miniconda*.

1. Download miniconda from https://www.anaconda.com/download.
2. Open the installer and follow its instructions to finish the installation.

***Note:*** For details on Anaconda installation, refer to https://www.anaconda.com/docs/getting-started/anaconda/install.

***Note:*** On Windows PCs, it may be necessary to run the command “*conda init cmd.exe*” before proceeding to step 3.

3. Once the process is complete, open the Anaconda Prompt to create a new virtual environment and activate it.

conda create -n forsys_env python=3.11

conda activate forsys_env

***Note:*** ForSys requires Python version 3.8 or later. In this example, we use Python version 3.11.

4. Install the latest stable ForSys version by using the “pip” Python package manager.
  pip install forsys

Methods video S1. Installing ForSys, A Conda environment is created, where ForSys is installed and run using the “-v” flag to check that it is working, related to steps 1–4

***Note:*** All information regarding the ForSys package in this article refers to ForSys in version 1.1.5. To install directly from source, or a specific version, please refer to the installation guide https://forsys.readthedocs.io/en/latest/installation.html.

### Preparing the input data and folder structure

**Timing: <10 min**

ForSys, by default, expects a folder structure in which each timepoint from live microscopy is an individual TIFF image, and each segmentation is contained in a folder with the same name as the corresponding image. The segmentation can be a TIFF skeleton or an NPY mask. If both are present, ForSys will use the TIFF skeleton.

5. Create a new directory on your computer where the input and output files will be placed. Place a “data” folder, where the input experiments will go, and a “results” folder for ForSys to save the inference outputs.

***Note:*** Please refer to the troubleshooting section for details on the expected folder structure.

***Note:*** The three modes to use ForSys (Command Line Interface, Graphical Interface and Scripting) will be described as “optional”, as any of the three should work in most cases, see Figure 1 for details. However, at least one of them must be followed.

### Using the command line interface

**Timing: 30 min**

We will now describe how to use the Command Line Interface to interact with ForSys. Offering more flexibility than the Graphical User Interface, the Command Line Interface is a good middle ground between writing custom Python scripts and the Fiji interface. All instructions from here on should be run in the operating system’s command line on your computer. Moreover, before starting this section, you should be inside the Python environment where ForSys was installed.

6. First, test that ForSys is correctly installed by running the following command.
  python -m forsys -v

Its output should be similar to “*ForSys version 1.1.5*”, with the numbers indicating the ForSys package’s version. If that is not the case, you can try running.

conda activate forsys_env

To make sure that you are in the correct environment.

***Note:*** If this does not work, repeat the steps in “preparing the Python environment”.

7. Running the inference is done in one single command, which changes between static and dynamic modalities. For the static modality, you should run.

python -m forsys -f /path/to/input_folder -m nnls -mt 3 -sf /path/to/save_folder

Where “/path/to/input_folder” should be replaced with the top-level folder where the images are (This should be the “data” folder described in section “preparing the Python environment”, starting on step 1), and “/path/to/save_folder” with the path to the folder where the results will be saved (This should be the “results” folder described in section “preparing the Python environment”, starting on step 1).

***Note:*** The other “flags” in the command are “-m”, to choose which solver to use and “-mt” to choose the maximum time for the inference; in this case, we only process the first three frames using the Non-Negative Least Squares method.^13^^,^^14^^,^^15^

**CRITICAL:** ForSys expects the folder with the inputs to contain one TIFF image per timepoint, followed by a folder with the same name as the TIFFs, which in turn contains a single file called “handCorrection”. The “handCorrection” file can be either a TIFF file or an NPY file, specifically from CellPose segmentations.^16^ If no “handCorrection” is found in the inner folder, it defaults to any TIFF image found in that folder, and then to any NPY mask found. Please refer to the troubleshooting section for details on the expected folder structure.

***Note:*** The previous command will run ForSys’s static modality on all the timeframes, regardless of having more than one timepoint.

***Note:*** The complete list of commands is available from the command line using the “-h” flag. For more information, refer to the documentation (https://forsys.readthedocs.io/en/latest/command_line.html).

8. For the dynamic modality, the command to run should be.

python -m forsys -f /path/to/input_folder -m nnls -mt 3 -sf /path/to/save_folder --dynamic -c /path/to/connections.json

***Note:*** The new flag “--dynamic” utilises a dynamic algorithm. The optional argument “-c /path/to/connections.json” specifies the location of the connections file and is optional. The connection file will be described in the section “correcting the automatic connections between timepoints (optional)”, starting on step 31. If the “-c” flag is not used, ForSys will use its own automatic connections without any corrections.

9. In any of the previously described modalities, inferred stresses and pressures can be exported by using the *-o* or *--output_csv* flags as.

python -m forsys -f /path/to/input_folder -m nnls -mt 3 -sf /path/to/save_folder --dynamic -c /path/to/connections.json -o

***Note:*** If microscopy images are available, ForSys can create a composite TIF image that superimposes the inference results on the experimental image. To do this, add the *-cc* or *--composite* to the commands shown in steps 7, 8 or 9. The experimental microscopies must be in the “*/path/to/input_folder*” folder.

### Installing the graphical user interface

**Timing: 15 min**

ForSys provides a Graphical User Interface implemented in Fiji^17^ to facilitate the inference. For a video tutorial of the installation steps, please see Methods video S2. To install the interface.

10. In Fiji, go to Help -> Update -> Manage update site.
11. Select “Add unlisted site” and add the URL: https://sites.imagej.net/ForSys
12. Select the ForSys update site and click “Apply and Close”, and then “Apply Changes”.
13. After restarting Fiji, you can open ForSys GUI by clicking Plugins -> ForSys Plugin.

Methods video S2. Installing the Graphical User Interface, ForSys graphical interface is implemented as a plugin in Fiji, related to steps 10–13

### Using the graphical user interface

**Timing: 30 min**

Here, we describe how to use the Graphical Interface after installing it in Fiji.

***Note:*** The graphical user interface provides quick and easy access to the main features of ForSys but offers less flexibility in its input and output. To remedy this, the GUI includes an “Extra arguments” textbox that can be used to add functionalities. These extra arguments can be found in the documentation (https://forsys.readthedocs.io/en/latest/command_line.html) and were also described in the section “using the command line interface”.

**CRITICAL:** Using the GUI requires a specific folder structure for the software to locate the input images, as described in the section “preparing the input data and folder structure”. Additionally, ForSys must be installed in the Python environment, and the “conda” command must be recognised by the default command line. Please see Methods video S3 for a tutorial on using the graphical interface.

14. Make sure the ForSys package has been installed, following the instructions in the section “preparing the Python environment”. To test this, you can follow step 6.
15. Open Fiji on your computer.
16. In the menu, select Plugins -> ForSys. You can also use Fiji’s search bar to find the plugin.
17. In the textbox “Conda Env”, write the name you gave to the conda environment created. If you followed the “preparing the Python environment” section, the name should be *forsys_env.*
18. Then, press “Browse” at the end of the first line (“Input folder”) and select the folder that contains the images to be analysed.

***Note:*** As mentioned in the section “using the command line interface” (step 7), the input images must have a specific folder structure, described in the note below step 7 and in problem 4 of the troubleshooting section.

***Note:*** In the section “correcting the automatic connections between timepoints”, starting on step 31, we will explain how to create a connections file to correct the timepoints created by ForSys. You can use the Browse button in the “Connection file” line to find the correct JSON file if you have one.

19. The “Results Folder” should be automatically filled. You can change the values to any folder you wish.
20. You can use the check boxes to add functionalities or change the appearance of the output. For example, the “Composite” checkboxes will output an image showing the inferred membrane stress in a colour map superimposed on the microscopy image, if the image is available in the input folder.
21. Press “Run ForSys” to run the inference.

***Note:*** To export the inference results as CSV files, please add “-o” or “--output_csv” to the field “Extra Arguments” below the output options in the GUI.

***Note:*** Please make sure that the decimal separator in the aspect ratio box corresponds to the one used in your local system (either comma or dot separated).

Methods video S3. Using the Fiji Plugin, ForSys Fiji plugin can be tested with the data available in the official repository, related to steps 14–18

### Importing *in silico* data through scripting

**Timing: 10 min**

In this section, we will import *in silico* data and load it into ForSys.

***Note:*** When running this portion of the protocol for the first time, we recommend using the synthetic data generated by seapipy^18^ and Surface Evolver^9^ that is provided in the “examples/data/in_silico” folder of the GitHub repository.^8^ The procedure is similar for both synthetic and experimental data, consisting of creating the ForSys object from the available frames.

**CRITICAL:** The conda environment must have ForSys installed, as described in the “preparing the Python environment” section.

***Note:*** Two implementations of this code, for the static and dynamic modality, can be found in Jupyter notebooks in ForSys’s GitHub repository, in the “examples/” directory, and in the package’s documentation (https://forsys.readthedocs.io/en).

22. Create a new directory as described in the last step of “preparing the Python environment” and a new Python file, such as “main.py”, where you will write the inference script. All the steps below involve writing the given code to the “.py” file created. In this way, you should have a directory with two subdirectories (“data” and “results”) and a Python file (“main.py”).

***Note:*** The following sections describe the software's use through Python scripting. To execute the software at each of these steps, you should use “python main.py” in the command line.

***Note:*** The following workflow can also be adapted for use in a Jupyter notebook, provided that it is installed in the environment. For this, a file with the extension “.ipynb” should be created. In this case, each step can be regarded as an individual “cell” in the notebook. For more information about Jupyter Notebooks, visit https://jupyter.org/.

23. Then you need to import all the libraries that will be used for the inference, with the corresponding input and output directories. The “DATA_FOLDER” and “RESULTS_FOLDER” values should be modified to have the route to your desired input and output folders, respectively.

import forsys as fs

import matplotlib.pyplot as plt

import numpy as np

import os

DATA_FOLDER = os.path.join("examples", "data", "in_silico")

RESULTS_FOLDER = os.path.join("results")

max_time = 5

if not os.path.exists(RESULTS_FOLDER):

  os.makedirs(RESULTS_FOLDER)

***Note:*** The outputs will be saved to a “results” folder, which will be created if it does not already exist. Only the first five frames will be inferred, as set by the “max_time” variable.

24. Then, you must read the synthetic data files to create the frame objects from the vertices, edges and cells data.

frames = {}

for time in range(max_time):

 if time == 0:

   real_time = 0

   elif time == 1:

   real_time += 1 ∗ 25000 ∗ 0.005

   else:

   real_time += 1 ∗ 50 ∗ 0.005

 lattice = fs.surface_evolver.SurfaceEvolver(os.path.join(DATA_FOLDER, f"step_{time}.dmp"))

frames[time] = fs.frames.Frame(time,

      lattice.vertices,

      lattice.edges,

      lattice.cells,

      time=real_time,

      gt=True)

***Note:*** The “real_time” variable allows ForSys to calculate the velocity of each vertex properly; in this case, using the examples found in the “examples/data/in_silico” folder of the repository, the values for the “real_time” variable represent the timesteps of the simulations and were described in the main paper.^1^ The “gt=True” option will also save the ground-truth stress values for each frame by reading the “densities” from the Surface Evolver output files.

25. Once this is done, the only thing needed is to pass all the frames to the ForSys class, so that it can be used for inferences. This step, and all steps hereafter, are the same for either synthetic or experimental data.
  forsys = fs.ForSys(frames)

### Importing *in vivo* data into ForSys through scripting

**Timing: 10 min**

In this section, we will show how to load time-lapse images into ForSys using Python scripting. The pipeline consists of reading the images, translating them into vertices, edges, and cells, creating the “frame” object from this data, then passing it to the ForSys class. The workflow between synthetic and experimental data differs only in how the frames are loaded. After the “forsys” object is created, there is no difference in the treatment of the frames. A description of how to use the Graphical User Interface and the Command Line is included at the beginning of the “step-by-step method details” section.

**CRITICAL:** A prerequisite for the following sections is that ForSys is installed in a conda environment, which we will call forsys_env, as described in the section “preparing the Python environment”.

***Note:*** Example Jupyter notebooks showcasing similar implementations to those described here can be found in ForSys’s GitHub repository and in the documentation (https://forsys.readthedocs.io/en).

26. Create a new directory for the project and a new Python file for the inference script. In the present protocol, we will refer to this file as main.py. Create also a folder to output all of the results information.

***Note:*** In the present protocol, it will be assumed to be in the same folder as main.py with “results” as the folder name. Therefore, the directory will have two subdirectories (“results” and “data”, and one file “main.py”).

***Note:*** Running ForSys via Python scripts enables high pipeline customisation. When using the graphical or command line interface, ForSys expects a specific folder structure for the input data. This is described in the corresponding sections.

27. From now on, you will modify the main.py file that was just created. First, import the necessary scripts.

import forsys as fs

import matplotlib.pyplot as plt

import numpy as np

import os

28. Set the input and output directories for ForSys to read from. We will assume that the microscopy data is in the same folder as the “main.py” file, in a subfolder called “data”, and that we only want to infer five frames.

DATA_FOLDER = os.path.join("data")

RESULTS_FOLDER = os.path.join("results")

max_time = 5

29. For each time point in the microscopy, create a “forsys.Frame()” object that will store the frame information. Each frame must be saved with consecutive integer numbering; however, the “time” argument can be used to specify the actual time of the experiment. If images have been taken every 30 seconds, the time argument could be 0.5 (half a minute).

frames = {}

for time in range(max_time):

  real_time = 1 ∗ time

  current_tif_file = os.path.join(DATA_FOLDER,

        f"t_{time}.tif")

  skeleton = fs.skeleton.Skeleton(current_tif_file, mirror_y=False)

  vertices, edges, cells = skeleton.create_lattice()

  vertices, edges, cells, _ = fs.virtual_edges.generate_mesh(vertices,

                edges,

                cells,

                ne=5)

  frames[time] = fs.frames.Frame(time,

                vertices,

                edges,

                cells,

                time=real_time)

30. Next, the frames must be passed to ForSys to preprocess them and generate the connections between consecutive times. These connections can be edited through a JSON file, which will be discussed in a separate section.
  forsys = fs.ForSys(frames)

***Note:*** The “forsys” object created in the last step of this section now has all the information regarding the system and will be the main point of interaction with the inference algorithm. A good practice to ensure the correct interpretation of the movements is to generate a plot to visualise the time connections of the vertices. How to plot the connections will be described in the section “correcting the automatic connections between timepoints”.

### Correcting the automatic connections between timepoints

**Timing: 1 day**

ForSys can automatically track vertices through time series. Sometimes it might be necessary to perform manual corrections of this tracking. In this section, we will show how to display the tracking information and correct any necessary connections. Once the microscopy data has been loaded into the ForSys software, it is good practice to plot the images to verify that the algorithm has correctly interpreted the segmentations and that the time connections of the vertices are correct. Moreover, plotting the mesh provides access to the IDs of the different elements, which may be necessary for further processing downstream. The most time-consuming step of this section is the manual correction of the connections between consecutive frames.

***Note:*** We have included an example Jupyter notebook in ForSys’s repository (in the “examples” folder, named “dynamic_in_vivo_with_connections.ipynb”) and in its documentation (https://forsys.readthedocs.io/en) that can be used to follow this section of the protocol.

To curate the vertex tracking, the best workflow is to create a separate Python file and repeat the steps shown in section “importing in vivo data into ForSys through scripting” up to step 30. From then on, continue through this section.

**Figure 2:**
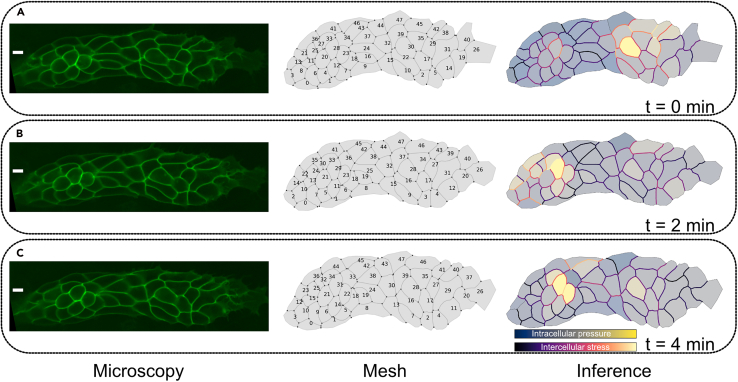
Mesh to inference Each panel from A-C shows a subsequent timestep, with images taken every 2 min. From left to right, we see the original microscopy image, the mesh generated by ForSys from the skeletonised segmentations, and the inference results. In the rightmost panel, the colour of the membranes represents the stress, and the colour filling of the cytoplasm represents the pressure. Pressure colourmaps have the following values, expressed as (minimum, maximum), for panels (A) (−0.67, 1.74), (B) (−0.70, 0.94) and (C) (−0.72, 1.16). The scale bar in the microscopy images, at the leftmost column, represent 5 um.

31. To plot the mesh, the module *plot* has the *plot_mesh* function. The elements to be drawn can be modified through different keyword arguments. In this example, it will draw the first timepoint with an inverted y-axis, displaying the IDs of all cells, the tricellular junctions with their corresponding IDs, and the vectors used in the force matrix calculation. An example of this, without the versors, can be found in the left column of Figure 2.

fs.plot.plot_mesh(frames[0],

     plot_cells_id=True,

     plot_tjs=True,

     plot_vertices_id=True,

     plot_versors=True,

     mirror_y=True)

***Note:*** For more information about the arguments that can be passed to the function, refer to the documentation at https://forsys.readthedocs.io/en/.

**Figure 3:**
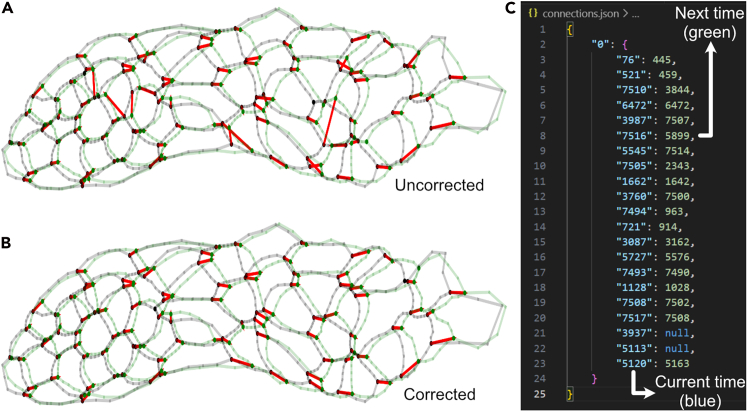
Creation of the connections file (A) Initial representation of the connections, before any correction is made. The black silhouette represents the current time point, while the green silhouette represents the next time point. Many vertices are correctly connected even before any user input is provided. (B) Result of connecting some of the vertices through a connection file. (C) Shows how the connections file is used to correct how panel (A) looks. In this case, out of 96 vertices, 20 had to be manually curated, as shown in panel (C).

32. The temporal connections between different tricellular junctions are estimated during the creation of the “forsys” object in step 30. To check its correctness, we can plot the connections from the initial (*t0)* to the final time (*tf)* and save them to a folder named “connections” in the results directory, as we defined in step 28. An example is shown in Figure 3A.

fs.plot.plot_time_connections(forsys.mesh,

      initial_time=0,

      final_time=len(forsys.frames),

      folder=os.path.join(RESULTS_FOLDER,

            "connections"))

33. If manually reviewing the generated plots reveals that some connections must be corrected, this can be done by using a JSON file. To this end, create a file in the working directory with the “.json” extension, for example, “connections.json”. This file will contain the ID of the vertices at time t, and their ID at time t+1 for each step t. The following is an example of how this JSON file might look. In this case, the vertex that has ID “1” at frame 0 will have ID “2” in the following frame, and vertex “3” at frame 0 will not exist at frame 1. Then, vertex ID “2” at frame 1 will still have ID “2” at frame 2. For a real application example, please see Figure 3.

{

"0":{

  "1": 2,

  "3": null,

  },

"1":{

  "2": 2,

  },

}

34. Now, to read the file and pass it to ForSys, use the script generated in section “importing in vivo data into ForSys through scripting”, and load the JSON file after step 29 using the “auxiliar” module with the path to the connections file, the initial frame and the last frame to load as,
  initial_guess = fs.auxiliar.load_initial_guess(path, t0, tf)

35. Then, modify step 30 by letting ForSys know of the “initial_guess” variable as
  forsys = fs.ForSys(frames, initial_guess=initial_guess)

36. After that, replot the connections as described in step 31. This workflow of plotting the connections and manually correcting them can be repeated until all connections are correctly assigned. The effect of the corrections is shown in Figure 3B.

***Note:*** It is seldom necessary to add all vertices to the connections file. Sometimes, adding a few vertices can guide the algorithm in the correct direction and substantially improve the connections. For example, using the file shown in Figure 3C, twenty corrections are enough to get an accurate representation of a system with around 100 vertices.

### Dynamic inference of the mechanical state

**Timing: 10 min**

After the ForSys object was created, as described in step 30 or step 35, depending on whether a manual correction was necessary, we generate the Force matrices and then let the algorithm solve the system of equations. As described in the main paper,^1^ in dynamic inference, the relationship between the elastic and viscous scales must be set. Thus, it is required to calculate the average velocity of the system to use in the scale parameter definition (ρ).^1^ A suggested method for calculating the scale parameter will be described in a separate section (Section “determination of the scale parameter (optional)”).

***Note:*** If it is not possible to measure the scale parameter, we recommend using a value of 0.1 (ρ = 0.1); for further information, see section “determination of the scale parameter (optional)”.

37. First, calculate the velocities of the system.

all_velocities = forsys.get_system_velocity_per_frame()

ave_velocity_to_normalize = np.mean(all_velocities)

vel_norm = 0.1 / ave_velocity_to_normalize

38. Then, create the matrix for the stresses, and solve the system. This is necessary to calculate the pressures afterwards, as pressure depends on membrane tension, according to the Young-Laplace equations.

for time in range(max_time):

  forsys.build_force_matrix(when=time)

  forsys.solve_stress(when=time,

      b_matrix="velocity",

      velocity_normalization=vel_norm,

      method="nnls",

      allow_negatives=False)

***Note:*** The “b_matrix” parameter in the “solve_stress” function can be used to create a static inference, that is, an inference that disregards the time evolution of the vertices. To perform a static inference, use “b_matrix=static” in the code above. A further description of static inferences is presented in section “static inference of the mechanical state of in vivo time-lapse microscopy”. More information about this at https://forsys.readthedocs.io/en/latest/examples/static_in_vivo.html.

39. Finally, solve the system to obtain the inferred pressures.

for time in range(max_time):

  forsys.build_pressure_matrix(when=time)

  forsys.solve_pressure(when=time,

       method="lagrange_pressure")

***Note:*** Other solver methods are available (lsq and lsqlinear). For more information, please refer to the documentation at https://forsys.readthedocs.io.

### Static inference of the mechanical state

**Timing: 10 min**

ForSys also allows performing static inferences. In this case, the inference results would be similar to other methods that use static algorithms, such as CellFIT^19^ and DLITE.^20^ Using a static modality usually requires less time, as the exact connections between time points are not necessary, allowing for a rapid exploration of the inference after experimental image acquisition. In those cases where only one frame exists, static inference is always used. The procedure is similar to what was described in section “dynamic inference of the mechanical state”, but the velocity calculations are not needed.

40. After step 24 for computational data, or step 29 for experimental data, create the force matrix, and solve the stress by using.

for time in range(max_time):

  forsys.build_force_matrix(when=time)

  forsys.solve_stress(when=time,

       method="nnls")

***Note:*** This will also work for the case where there is only one time point (max_time = 1).

41. After solving for the stress, you can create and solve the pressure matrix the same as before.

for time in range(max_time):

  forsys.build_pressure_matrix(when=time)

  forsys.solve_pressure(when=time,

        method="lagrange_pressure")

### Determination of the scale parameter

**Timing: 1 week**

The scale parameter (ρ) is an important element of the dynamic inference, as it captures the relationship between the viscous and elastic scales of the system under study.^1^ We have previously proposed a value of 1/10 (ρ = 0.1), but it is possible to experimentally determine this parameter by performing *in vivo* laser ablation experiments in tissues of the same type.^21^ A more detailed description, as well as the application of this protocol’s section to the zebrafish lateral line neuromasts, can be found in Borges et al.^1^ The following steps require the use of a sample with fluorescently tagged membranes for segmentation.

***Note:*** To avoid affecting the system's recoil dynamics, only one membrane should be ablated per tissue.

***Note:*** Several recoil events should be recorded to provide a better determination of the scale parameter.

42. Record live imaging of the tissue of interest that includes the moments before, during and after the ablation of one membrane in the tissue (Figures 4A and 4B).

**Figure 4:**
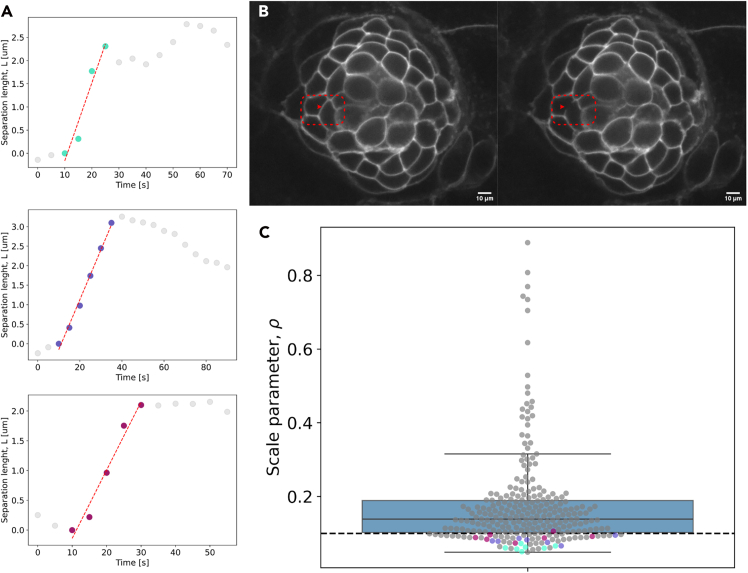
Determination of the scale parameter through ablation experiments The scale parameter is determined from the linear fits of the separation length after laser ablation of a cell membrane in the neuromast organ. (A) shows three example trajectories and the corresponding linear fits. Panel (B) is shown as an example of the laser ablation experiment, corresponding to the topmost trajectory in (A). (C) shows the distribution of values of the scale parameter for a whole set of ablation experiments (N=37) from the experiments performed to determine the average velocity (N=7). The orange dots in panel (C) highlight the 21 (3x7) points corresponding to the three experiments in panel (A). Scale bars in panel (B) represent 10 um each.
43. Track the distance between the two tips of the ablated membrane in time.
44. Fit the trajectory to Equation 1 of Borges et al.,^1^ i.e.,

(Equation 1) $$L\left(t\right)=L_{0}+2\;\alpha \;t\text{.}$$

Where *L*_0_ represents the initial length, and *α* (=*λ*/*η*) is the ratio between membrane stress and viscous damping coefficient. Three example trajectories with the corresponding linear fit to Equation 1 are shown in Figure 4A. One example of ablation is shown in Figure 4B, in the neuromast of the zebrafish’s lateral line, corresponding to the first trajectory in panel A.

***Note:*** The fit should be performed to the datapoints that describe the movement. Depending on the data acquisition frequency and the system dynamics, the fitting interval has to be adjusted.

45. Segment the cell membranes in the live imaging movies up until the ablation frame. The average velocity of the system can be calculated using ForSys as described in step 37.
46. The linear fit suggested above will provide a value for *α* in each experiment. This value can be used to obtain the Scale parameter as.

(Equation 2) $$\rho =\frac{\bar{v}}{\alpha }\text{.}$$

Where $\bar{v}$ is the average velocity of the system, described in step 38.

***Note:*** It is possible to use the average velocity of the system, as determined by other experiments in which the tissue is not ablated. In this way, there will be a scale parameter *ρ* for each ablation (where the *α* was determined) and for each non-ablated experiment (where the average velocity $\bar{v}$ was determined), see Figure 4C.

47. The scale parameter to be used in the inferences can be obtained by using the mean or median of the distribution of scale parameters (*ρ*) calculated for each ablation experiment. The mean value obtained would replace the 0.1 used at step 37 (Figure 4C).

### Plotting the inference results

**Timing: 15 min**

The system’s results can be displayed in several ways. In this section, we will show how to display the intercellular stresses and intracellular pressures as colour maps. For a reference on how to export the data for further analysis, please refer to the section “quantifying the inference results”.

48. The plot module has the “plot_inference()” function, which handles the creation of each frame’s image. For example, to plot the mechanical state of frame *time*, you should use.

fs.plot.plot_inference(forsys.frames[time],

      normalized="max",

      colorbar=False,

      pressure=True,)

plt.savefig(os.path.join(RESULTS_FOLDER,

      f“time_{time}.png”),

    dpi=300)

***Note:*** This example creates the image for the frame “time”, normalising to the maximum stress (normalized=“max”) and excluding the colorbar (colorbar=False). To include the colorbar, the parameter colorbar must be true (“colorbar=True”). As “pressure=True” was included, the inferred pressures will also be included in the plot as a colour map inside the cells.

***Note:*** A composite image that superimposes the inferred stress as a colour map on the experimental microscopies can be created using the plot_inference_as_tiff() function of the plot module. This function takes as input the ForSys object, the image sizes, and the folder where the images should be saved. To include experimental microscopies, they must be passed to the function via the “original_images” keyword argument. If this argument is not used, a TIFF sequence with just the inferred stress will be generated.

***Note:*** For other normalisation options (“normal”, “relative”, and “absolute”), please refer to the documentation at https://forsys.readthedocs.io.

***Note:*** An example of the execution of the code above is shown in Figure 2, right column.

### Quantifying the inference results

**Timing: 15 min**

Getting a quantitative description is also possible with ForSys. The values for the intercellular stresses and intracellular pressures can be accessed as a Pandas dataframe,^22^^,^^23^ stored in the Frame objects.

49. To access stress and pressure values, you can use the *get_tensions()* and *get_pressures()* functions after the system has been solved. For example, to get stress and pressure distributions for the first step, use.

tensions_dataframe = forsys.frames[0].get_tensions()

pressures_dataframe = forsys.frames[0].get_pressures()

50. Additionally, the create_csvs() function, in the auxiliary module, can be called to create two dataframes with additional information about the cells and membranes in the tissue. This function needs the frame for which the information should be exported. Then you can save using DataFrame.to_csv() in the Pandas package. For example, in the case of frame 0,

cell_df, stress_df = fs.auxiliar.create_csvs(forsys.frames[0])

cell_df.to_csv(“cell_info.csv”)

stress_df.to_csv(“membrane_info.csv”)

***Note:*** The cell_df dataframe will have columns for ID, area, perimeter, centroid position, and inferred pressure for all cells in the tissue (called id, area, perimeter, position_x, position_y, and pressure). The force_df dataframe will contain the ID, stress, length, centroid, and curvature of all the membranes (called: id, tension, length, position_x, position_y, and curvature).

## Expected outcomes

Using this protocol, you will generate representations of the mechanical state of the system in the form of plots where the cell membranes are colour-coded by mechanical stress, and the cell’s interior by intracellular pressure (Figure 2, right panel). The two auxiliary functions described in steps 49 and 50 will also give numerical values for stress and pressure. The numerical values of stress are expressed relative to the average value.

## Limitations

The method’s main limitation is its reliance on the quality of the input data. This encompasses both the fidelity of the segmentations and the accurate tracking of the changes in the vertices’ positions over time. Movements generated by agents other than the cell in the region of interest will not be captured by the algorithm, potentially leading to confounding effects. A more thorough discussion of the limitations of this method and stress inference in general has been discussed elsewhere by other authors as well as by us.^1^^,^^2^^,^^7^

## Troubleshooting

### Problem 1

Anaconda gives an error saying “Conda not recognised”.

### Potential solution

If you are using Anaconda and encounter the error “Conda not recognised”, it typically means that the Anaconda installation path is not added to your system’s PATH environment variable. For more info, follow the Anaconda installation guide (https://docs.conda.io/projects/conda/en/stable/user-guide/install/index.html).

### Problem 2

There is an error “ModuleNotFoundError: No module named ‘forsys'”.

### Potential solution

Make sure you are in the correct environment and that ForSys is properly installed. Repeat steps 1 to 4 if necessary.

### Problem 3

I get an error that says “ValueError: max() arg is an empty sequence”.

### Potential solution

You have probably not run the inference solver. Please make sure that you are running all the steps described in either “dynamic inference of the mechanical state” (step 37) or “static inference of the mechanical state” (step 40).

### Problem 4

Using the Graphical Interface (GUI) or the Command Line (CLI), I receive an error message and the text: “Found 0 frames in the system.” Is in the output.

### Potential solution

Most likely, the input segmentations are not being found by the algorithm. Please first verify that the input folder selected through the GUI or via the “-f” flag in the CLI is correct. If this is the case, ensure that each segmentation is in a separate subfolder. For example, if the input images are in the data/folder, the structure should be:

data/

step_1.tif

step_2.tif

step_1/

segmentation.tif

step_2/

segmentation.tif

The TIFF images at the top will be regarded as experimental images, and the ones inside the subfolders will be considered the segmentation masks. These masks can either be.tif skeletons or CellPose binary masks (.npy format).

### Problem 5

My inference shows very high stress in a few edges, and almost zero in the rest.

### Potential solution

Stress inference techniques, such as ForSys, rely on a segmentation that faithfully represents the system. This typically indicates that the segmentation contains geometries that may be problematic for the algorithm. For example, short edges, wavy cellular interfaces and T-like tricellular junctions could lead to issues. Sometimes, it is possible to find a different z-plane in which some of these problems are mitigated.

### Problem 6

I was able to run the inference, but I can’t find a way to export the numerical values generated by ForSys.

### Potential solution

Depending on the method you use to run ForSys, the steps for exporting numerical values might vary. If you are using the Graphical User Interface, you can write --output_csv (or -o) in the “Extra Arguments” field in ForSys’ plugin. In the Command Line, please, write --output_csv (or -o) at the end of the command you are running. Finally, in Python scripting, you can access the same information using the auxiliary module to run.

cell_df, force_df = fs.auxiliar.create_csvs(forsys.frames[time])

The cell_df variable will contain the ID, area, perimeter, centroid position and inferred pressure of all cells in the tissue, and the force_df variable will have the ID, stress, length, centroid and curvature of all the membranes.

### Problem 7

When I run the inference, I get errors similar to “ValueError: zero-size array to reduction operation maximum, which has no identity” or others that include some reference to the NumPy package.

### Potential solution

This type of error occurs when ForSys can’t correctly interpret the segmentation that was used as input. The most common source of this error is the existence of cells that touch the border, which ForSys can’t handle. To solve it, check that the segmentation includes only full cells (i.e., no cells whose boundaries are the edge of the microscopy).

If there are no cells in the border, this error can also occur when ForSys is unable to reconstruct the tissue from the segmentation. ForSys deals successfully with many potential sources of segmentation artefacts under the hood, but there are corner cases that might not work. When this happens, we recommend curating the segmentation: sometimes, membranes and triple junctions that are problematic can be directly observed. If none of this works, please get in touch with us by email or via the GitHub repository’s issue tracker so we can help resolve the issue for your individual case.

***Note:*** For more information on troubleshooting, please visit ForSys documentation (https://forsys.readthedocs.io/en/latest/faq.html). If your case is not included in the documentation, you can always reach out through the GitHub Issues tracker (https://github.com/borgesaugusto/forsys/issues).

## Resource availability

### Lead contact

Further information and requests for resources should be directed to and will be fulfilled by the lead contact, Osvaldo Chara (osvaldo.chara@nottingham.ac.uk).

### Technical contact

Technical questions on executing this protocol should be directed to and will be answered by the technical contact, Augusto Borges (ab3330@cam.ac.uk).

### Materials availability

This study did not generate any new materials.

### Data and code availability

• The seapipy codebase is available on GitHub at https://github.com/borgesaugusto/seapipy and on Zenodo.^18^
• ForSys is available on GitHub (https://github.com/borgesaugusto/forsys) and on Zenodo.^8^
• Example notebooks are available at https://github.com/borgesaugusto/forsys/tree/main/examples.
• ForSys documentation, including installation instructions, is available at https://forsys.readthedocs.io/en/.

## Acknowledgments

A.B. acknowledges support through a Research Project Grant from the RPG-2025-081 10.13039/501100000275Leverhulme Trust (awarded to Prof. Ewa Paluch). A.S.C. and O.C. were funded by a 10.13039/501100000268BBSRC grant BB/X014908/1 to O.C., O.C. was also funded by UADE grants A23T01 and P26T02 to O.C. J.S. acknowledges the support of the 10.13039/100032285Novo Nordisk Foundation (grant nos. NNF22OC0076414 and NNF19OC0056962), the 10.13039/501100012331LEO Foundation (grant no. LF-OC-19-000219), and the 10.13039/100010663European Research Council Consolidator Grant (ERC CoG 101125803 MechanoFate). The Novo Nordisk Foundation Center for Stem Cell Medicine (reNEW) is supported by Novo Nordisk Foundation grant no. NNF21CC0073729.

## Author contributions

A.B. wrote the manuscript. A.B., A.C., and J.R.M.-R. tested the protocol. A.B., J.R.M.-R., A.C., G.V., J.S., H.L.-S., and O.C. edited the manuscript.

## Declaration of interests

The authors declare no competing interests.
